# Implementing care coordination in a large dental care organization in the United States by upskilling front office personnel

**DOI:** 10.1186/s12960-021-00593-0

**Published:** 2021-04-07

**Authors:** Aubri M. Kottek, Kristin S. Hoeft, Joel M. White, Kristen Simmons, Elizabeth A. Mertz

**Affiliations:** 1 Department of Preventive and Restorative Dental Sciences, School of Dentistry, University of California, San Francisco, 490 Illinois Street, Floor 11, Box 1242, San Francisco, CA 94143 United States of America; 2 Healthforce Center at UCSF, School of Dentistry, University of California, San Francisco, 490 Illinois Street, Floor 11, Box 1242, San Francisco, CA 94143 United States of America; 3 Willamette Dental Group, P.C., 6950 NE Campus Way, Hillsboro, OR 97124 United States of America; 4 Skourtes Institute, 6950 NE Campus Way, Hillsboro, OR 97124 United States of America

**Keywords:** Case management, Implementation science, Evaluation studies, Health care quality, access, and evaluation, Dental staff, Dental care, Care coordination

## Abstract

**Background:**

Care coordination is a key strategy used to improve health outcomes and efficiency, yet there are limited examples in dentistry. A large dental accountable care organization piloted care coordination by retraining existing administrative staff to coordinate the care of high-risk patients. Following the pilot’s success, a formal “dental care advocate” (DCA) role was integrated system-wide. The goal of this new role is to improve care, patient engagement, and health outcomes while integrating staff into the clinical care team. We aim to describe the process of DCA role implementation and assess staff and clinician perceptions about the role pre- and post-implementation.

**Methods:**

Guided by the Consolidated Framework for Implementation Research, semi-structured interviews with clinical and operational administrative staff and observation at the company-wide training session were combined with pre- and post-implementation electronic surveys. Descriptive statistics and mean scores were tested for significance between each survey sample (t-tests), and qualitative data were thematically analyzed.

**Results:**

With preliminary evidence from the pilot and strong executive support, a dedicated leadership team executed a stepwise rollout of the DCA role over 6 months. Success was facilitated by an organizational culture of frequent interventions deployed rapidly through a centralized system, along with supportive buy-in from managerial teams and high staff acceptance and enthusiasm for the DCA role before implementation. Following implementation, significant changes in attitudes and beliefs about the role were measured, though managers held stronger positive impressions than DCAs. DCAs reported high confidence in new skills and dental knowledge post-implementation, including motivational interviewing and the ability to confidently answer patients’ questions about their oral health. Overall, the fast-paced implementation of this new role was well received, although consistent and significant differences in mean attitudes between managers and DCAs indicate more work to fine-tune the role is needed.

**Conclusions:**

Successful implementation of the new DCA role was facilitated by a strong organizational commitment to team-based dentistry and positive impressions of care coordination among staff and managers. Upskilling existing administrative staff with the necessary training to manage some high-risk patient needs is one method that can be used to implement care coordination efforts in dentistry.

**Supplementary Information:**

The online version contains supplementary material available at 10.1186/s12960-021-00593-0.

## Background

Oral diseases are closely associated with several other chronic diseases [1, 2] and impact all aspects of health over the lifespan (including social, emotional, physical, and economic) [3], yet they are among the most significant unmet health needs in the world [4–8]. Oral diseases rank in the top ten leading causes of years lived with disability globally [9], with treatment costs estimated to be an average of 4.6% of total global health expenditures in 2010 and combined direct and indirect costs estimated near $442 billion USD annually [10]. The populations most prone to these diseases are also the most vulnerable: the poor, the very young, the elderly, those with disabilities, and those with comorbidities [4, 11]. Due to a myriad of reasons, dental care is underutilized in these high-risk, underserved populations and broken appointments are a common concern for both patients and providers [12].

Greater efforts and different strategies are required to meet current global oral health needs among high needs populations. Care coordination is a strategy increasingly used in health care to help meet the needs of vulnerable populations, but it has not been widely adopted in the dental field. In the United States (US), using a care coordination approach to dental care was explored by the American Dental Association in 2004 [13], resulting in a large pilot to train and implement Community Dental Health Coordinators [14]. Other models that have been used for care coordination in dentistry so far include case managers, community health workers with specific training in oral health, or social workers, all requiring hiring or bringing in an additional provider to take on the role of care coordination, which can pose financial or logistical barriers for dental practices. Evidence on programs that currently exist are focused on increasing utilization for a particular vulnerable population (e.g., patients with HIV/AIDS or public insurance), helping patients complete treatment, or improving referrals between medicine and dentistry [15–25], but very rarely do studies provide thorough information on how care coordination was implemented.

Existing dental team members already ubiquitous in dental practices, front office personnel, could be an untapped resource trained to provide care coordination. Office staff generally share similar demographics and languages with their patients, are someone patients are used to interacting with, and therefore may be more approachable or relatable to patients [26]. This strategy was deployed by the Willamette Dental Group (WDG): upskill office staff into care coordinators titled “dental care advocates” (DCAs) to bridge any gaps between patients and providers, improve attendance, facilitate treatment plan completion, and provide patient education. The rationale is to maximize use of existing staff and to create a more cohesive patient-centered team across traditional domains of front (administrative) and back (clinical) staff.

In 2015, WDG participated in a pilot project to improve integration of medical and dental care for patients with diabetes and/or tobacco use in response to Oregon’s Medicaid payment innovations incentivizing care coordination [27]. WDG’s central training division developed an initial training module with three core tenets: oral health knowledge, medical-dental knowledge, and motivational interviewing. Front office staff from three practices in Oregon were selected to participate. These newly trained DCAs were deployed at the beginning of January 2016, and by March, the executive team sought to scale the care coordination intervention company-wide. Evidence from the one-year intensive care coordination pilot supported their decision: 37 WDG patients participated, resulting in significant annual cost savings and fewer total medical visits compared to a control group of similar individuals [28].

To our knowledge, there is no similar example of a transformation of staff roles in the US dental delivery system, implementing care coordination by upskilling front office personnel, and no detailed account of the internal dynamics, implementation process, and dental team’s perspectives on this kind of workforce innovation. The aims of this mixed methods study are to describe the role, implementation climate, and process WDG undertook to roll out this new role company-wide, and to assess staff attitudes, beliefs, and confidence both before and 5 months after full implementation.

## Methods

### Study setting

WDG is a large multi-specialty group practice with more than 1,400 employees and 50 clinical practice sites across Oregon, Washington, and Idaho with approximately 500,000 patient visits per year. As a dental accountable care organization, they have focused on prevention since their founding in 1970. They have a system-wide electronic health record (EHR) system with imbedded clinical decision support tools for Caries Management by Risk Assessment (CAMBRA) and Periodontal Management by Risk Assessment (PEMBRA) [29, 30], clinical guidelines based on an individual’s risk for disease [31, 32]. WDG’s target populations for care coordination are individuals at high risk for caries or periodontal disease. Approximately a fifth of the patients in the WDG system are at high or extreme risk for caries and around a tenth are at high or extreme risk for periodontal disease [33].

### Study design

**Fig. 1:**
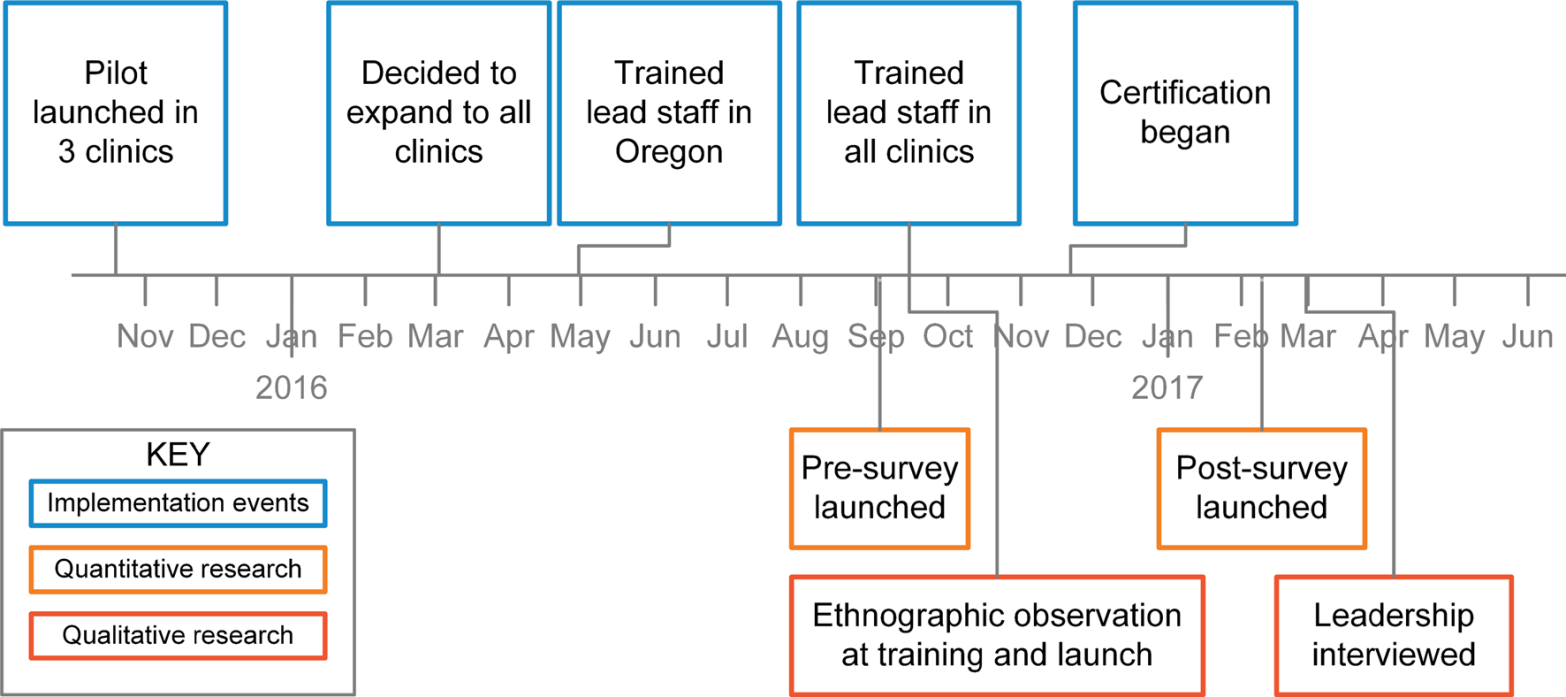
Timeline of implementation and research events

The Consolidated Framework for Implementation Research (CFIR) was used to guide our mixed methods design for systematically assessing the DCA implementation across five domains: (1) inner and (2) outer settings, (3) process, (4) intervention characteristics, and (5) characteristics of individuals [34]. Data sources included interviews, training session observation, and pre- and post-implementation surveys of attitudes and perceptions of the DCA role (Fig. 1).

The WDG administrators and leaders were interviewed by phone as a group to understand the intervention, its context, and the implementation plan following a semi-structured interview guide. A participant observation field guide was developed to systematically observe the formal launch of the intervention at the company-wide training session, including details on presentations (who, what, how, questions and answers), settings, observations by role, and reactions and interactions annotated each half hour.

Pre- and post-implementation electronic surveys were administered to practice staff and were modeled on prior surveys used to assess the implementation of clinical decision support tools at WDG [29, 35]. The surveys measured knowledge, opinions, and beliefs about the new DCA role and its implementation, began with questions clarifying job title and tenure, and concluded with demographic questions. Questions specific to the intervention and DCAs’ confidence in new skills were developed based on the WDG senior leadership’s goals for the role and the training they received. Additional items evaluated individual and practice efficacy and effectiveness, general job satisfaction, and experiences working at WDG. Most items were rated on a 5-point Likert scale. At the end of each survey, an optional open field collected comments about the implementation.

WDG provided a complete list of their employees at each of their dental practices, including job titles and email addresses, to send secure and confidential links to the surveys via email. The anonymous pre-implementation survey was provided as voluntary pre-work to the company-wide training on the new DCA role. Only employees attending this training were invited to complete the pre-implementation survey: each clinic’s managing dentist, practice manager, and lead front office staff. The post-implementation survey was launched 5 months later and was sent to the same group of managing dentists and practice managers, but all front office personnel (now “DCAs”), not just leads, were surveyed in the post-implementation survey. Across both surveys, 358 unique individuals were invited to participate. Study data were collected and managed using REDCap electronic data capture tools hosted at the University of California, San Francisco [36]. REDCap is a secure, web-based application designed to support data capture for research studies, providing (1) an intuitive interface for validated data entry; (2) audit trails for tracking data manipulation and export procedures; (3) automated export procedures for seamless data downloads to common statistical packages; and (4) procedures for importing data from external sources.

Qualitative data were thematically analyzed using the CFIR framework. Descriptive statistics were computed for each survey item in both samples, with mean attitude scores compared using 2-tailed tests of significance (alpha = 0.05). Paired analyses were conducted for respondents who completed both surveys. Sub-analyses were conducted stratifying mean attitude scores by respondent demographics and job types. Managing dentists and practice managers were grouped as “managers”, separate and distinct from DCAs in their supervisory and administrative roles. Stata 13.1 statistical software (StataCorp LP; College Station, TX) was used.

## Results

### Intervention characteristics

WDG’s goal was to transform and empower front office personnel into what they are now calling “DCAs”. DCAs are designed to be an integral member of the dental care team, providing documented case management notes in the patient’s EHR after each interaction and rallying the team when their patients return to the clinic. WDG upscaled the position with a compensable wage range to attract and retain talent. In addition to maintaining their front office roles and responsibilities (checking patients in/out, insurance and product transactions), the DCAs were trained in basic clinical dental knowledge and skills needed to manage their high-risk patients. These included motivational interviewing, helping patients to understand and follow through with recommended prevention and treatment services, how to receive and properly use prescriptions and home care products, scheduling recall appointments, and troubleshooting any barriers the patients might face. DCAs are tasked with conducting follow-up and personal reminder calls with high-risk patients in their practice as well as active outreach calls to patients who have not been to the clinic. These duties require DCAs to have a trusting and engaged relationship with the patient.

### Implementation process

Following the pilot’s success, full-scale implementation was planned as a phased rollout over 6 months (Fig. 1), starting with the lead front office staff at each of the remaining offices in Oregon trained in May 2016. The DCA training program is built on the 70–20–10 model of learning: 70% is on-the-job experiential learning, 20% through coaching/mentoring, and 10% is structured coursework.

The rollout was designed to be flexible to allow space for feedback and each office to adapt the role to their specific clinical environments. With feedback and guidance from the first three DCAs, this training included the same content and skills-based exercises, but instead of clear-cut new roles and responsibilities, these staff were tasked with figuring out the best way to implement the new role within their teams (i.e., the train-the-trainer method). This new approach of vaguely defining the role and then asking the offices to test the intervention was a change from the typical top-down culture the staff were accustomed to. The mixed approach of executive direction with flexible implementation in each office was difficult for some offices that were used to and even preferred the top-down approach. Recognizing these difficulties, WDG quickly decided to train all lead front office personnel in each of the three states in a formal launch of the role at a company-wide training in September 2016.

This training included all lead front office staff and managers, and again focused on clinical dental knowledge and skills-based exercises with a heavy emphasis on patient engagement. These newly trained DCAs and their managers were then taught the next steps to diffuse the implementation within each practice and train the remaining front office personnel. Regional refresher trainings were held about a month later to support each practice’s implementation of the role. WDG administrators developed a certification exam, case presentation, and competency matrix to standardize training and competencies across all DCAs. DCAs were tested by their managing dentists and certified from November–December 2016. Some offices and staff took on the role immediately, while others needed more time to figure out workflows, re-train each staff member, and evaluate and adjust the rollout to suit their practice demands.

### Characteristics of individuals

The pre- and post-implementation surveys provided an opportunity to assess individuals’ knowledge, attitudes, values, self-efficacy, and other personal attributes related to the implementation of the DCA role. Table 1 details the response rates to the pre- and post-survey by job type. The distribution of survey respondents by job type was consistent, though nearly four times as many DCAs were surveyed and responded to the follow-up survey. Of the baseline survey respondents, 63.3% (*n*  = 76) also responded to the post-implementation survey. Table 2 provides demographic characteristics of the matched pre/post respondents. The DCAs were all female and are far younger and more racially/ethnically diverse than the managers, though the practice managers were also more female (83.3% vs. 33.3%) and younger (61.1% < 45 years old vs. 33.3%) than the managing dentists. Managing dentists reported the longest tenure, whereas 63.9% of practice managers and 72.7% of DCAs reported less than 10 years of experience.

**Table 1:** Response rates by job type among each survey sample

Survey respondents	Managing dentists (*n*)	Practice managers (*n*)	Dental care advocates (*n*)	Overall response rate (%)
Baseline (pre)	30	43	32^a^	69.0%
Follow-up (post)	25	40	117	61.3%
Matched (pre/post)	18	36	22	63.3%

^a^Only lead front office staff at each office were invited to the company-wide training and surveyed at baseline; all front-office-staff-turned-DCAs were surveyed at follow-up

**Table 2:** Demographics of the matched pre/post-survey respondents

Respondent demographics	Managing dentists (*n* = 18), %	Practice managers (*n* = 36), %	Dental care advocates (*n* = 22), %
Gender			
Female	33.3	83.3	100
Age			
35 or younger	0	22.2	40.9
36–45	33.3	38.9	27.3
46–55	22.2	22.2	9.1
56 or older	16.7	8.3	9.1
Declined to answer	27.8	8.3	13.6
Race/ethnicity			
Non-Hispanic White	72.2	80.6	72.7
Hispanic/Latino/a/x	0	5.6	13.6
Asian/Pacific Islander	16.7	2.8	4.5
Black/African American	0	2.8	0
Another identity	0	2.8	0
Declined to answer	11.1	5.6	9.1
Years in dental field			
5 or fewer years	0	36.1	40.9
6–10 years	11.1	27.8	31.8
11–15 years	44.4	8.3	9.1
16–20 years	11.1	13.9	13.6
More than 20 years	33.3	13.9	4.5

Before implementation, there was a high level of excitement [mean = 4.50, standard deviation (SD) = 0.79] and support (mean = 4.56, SD = 0.72) for the DCA role. Respondents universally rated all items at least neutral (i.e., 3), with the lowest being the perceived ease of implementation (mean = 3.56, SD = 0.96; see Additional file 1 for full pre- and post-survey results). Staff felt strongly that the DCA role could improve communication with patients (mean = 4.66, SD = 0.59) and help patients feel more supported (mean = 4.64, SD = 0.62). Staff felt confident in their abilities to adapt to change (mean = 4.65, SD = 0.55), but were less confident in their team’s ability (mean = 4.01, SD = 0.76). Staff were engaged and energetic throughout the company-wide training, especially during role-playing activities and training sections focused on patient engagement.

Matched pre/post respondents generally held positive beliefs about the role before implementation, and these beliefs were strengthened afterward for things like their understanding of the role (mean difference =  + 0.59, *p* < 0.0001), the importance and value of the role (mean difference = + 0.16, *p* = 0.027), and whether it could improve patients’ oral health (mean difference = + 0.19, *p* = 0.036) (Table 3). Items that declined in mean scores included the perceived adaptability of the role (mean difference = − 0.14, *p* = 0.21) and how difficult it was to implement the role (mean difference = − 0.17, *p* = 0.29).

**Table 3:** Differences in attitudes and beliefs between survey respondent groups

	Matched pre/post sample (*N* = 76)				Follow-up (post) sample comparing dental care advocates to managers (*N* = 182)	
	Baseline (pre) mean (SD)	Follow-up (post) mean (SD)	Mean difference		Managers (*n* = 65), mean (SD)	Dental care advocates (*n* = 117), mean (SD)
Attitudes and beliefs about the new care advocate role						
You understand the role of the care advocate	4.20 (0.80)	4.79 (0.41)	+ 0.59***		4.85 (0.36)	4.21 (0.77)***
The investment in care coordination is worthwhile	4.54 (0.74)	4.70 (0.57)	+ 0.16*		4.74 (0.54)	4.03 (0.92)***
You believe implementing the care advocate role is important	4.62 (0.68)	4.76 (0.49)	+ 0.14		4.86 (0.35)	3.96 (1.04)***
Implementing care coordination positions WDG to adapt to a changing health care environment	4.41 (0.89)	4.62 (0.57)	+ 0.22*		4.63 (0.57)	4.02 (0.88)***
Implementing care coordination provides WDG a competitive edge in the dental marketplace	4.36 (0.92)	4.57 (0.67)	+ 0.21		4.57 (0.69)	3.99 (0.89)***
You are excited about the care advocate role	4.54 (0.74)	4.66 (0.67)	+ 0.12		4.75 (0.59)	3.76 (1.23)***
There is sufficient scientific evidence to support implementing care coordination in dentistry	3.92 (0.99)	4.38 (0.73)	+ 0.46**		4.41 (0.74)	3.73 (0.90)***
The goals of the care advocate role were clearly communicated^a^					4.32 (0.83)	3.72 (1.09)***
The care advocate role will be/was able to be adapted to your specific clinical environment	4.26 (0.60)	4.13 (0.79)	− 0.14		4.20 (0.86)	3.64 (0.96)**
The care advocate role has been working well in the practice where I work^a^					4.20 (0.96)	3.62 (0.99)**
Training and/or preparation for the care advocate position was sufficient^a^					4.22 (0.84)	3.57 (1.19)***
I was given an opportunity to provide feedback on the care advocate role^a^					4.09 (1.08)	3.53 (1.25)*
Integrating the care advocate role into your practice was difficult^b^	3.71 (0.91)	3.54 (1.15)	− 0.17		3.61 (1.12)	3.42 (1.10)
Beliefs about the new role’s impact						
Improve communication with patients	4.67 (0.58)	4.69 (0.57)	+ 0.03		4.77 (0.52)	4.23 (0.71)***
Help patients feel more supported	4.65 (0.56)	4.64 (0.61)	− 0.01		4.71 (0.58)	4.18 (0.72)***
Improve team communication about patient care	4.45 (0.62)	4.51 (0.71)	+ 0.07		4.57 (0.71)	3.94 (0.88)***
Enhance the effectiveness of clinical care	4.39 (0.82)	4.44 (0.68)	+ 0.05		4.52 (0.66)	3.95 (0.87)***
Improve the quality of dental care	4.38 (0.91)	4.44 (0.75)	+ 0.07		4.49 (0.69)	3.97 (0.91)***
Improve patients' oral health	4.30 (0.77)	4.49 (0.76)	+ 0.19*		4.52 (0.74)	3.93 (0.93)***
Improve patient compliance with provider recommendations	4.30 (0.79)	4.48 (0.78)	+ 0.18*		4.53 (0.78)	3.89 (1.01)***
Improve practices' productivity and efficiency	4.17 (0.90)	4.25 (0.80)	+ 0.08		4.35 (0.74)	3.58 (1.06)***
Improve workflow in my practice	4.00 (0.97)	4.16 (0.96)	+ 0.16		4.37 (0.80)	3.41 (1.15)***
Efficacy and general work satisfaction						
Please rate your confidence level in your personal ability to adapt to change	4.64 (0.56)	4.59 (0.59)	− 0.05		4.72 (0.48)	4.32 (0.74)***
Please rate how well you feel your personal values are aligned with the values of the organization	4.59 (0.59)	4.62 (0.59)	+ 0.03		4.69 (0.56)	4.21 (0.80)***
All in all, how satisfied would you say you are with your job?	4.43 (0.70)	4.63 (0.67)	+ 0.20*		4.72 (0.65)	4.01 (0.91)***
Please rate how well you feel integrated in the oral health care team	4.23 (0.75)	4.35 (0.71)	+ 0.12		4.45 (0.66)	3.89 (0.79)***
Please rate the extent to which you feel you have adequate resources and support to get your work done	3.91 (0.79)	4.12 (0.77)	+ 0.21*		4.20 (0.73)	3.87 (0.80)*
Please rate your confidence level in your team's ability to adapt to change	4.00 (0.77)	4.05 (0.76)	+ 0.05		4.25 (0.64)	3.84 (0.80)***
Please rate how well you feel your current skills are being utilized within the organization	4.01 (0.86)	4.24 (0.83)	+ 0.22*		4.38 (0.72)	3.71 (1.03)***
Please rate the current effectiveness of communication within your practice/team	4.13 (0.66)	4.09 (0.77)	− 0.04		4.14 (0.73)	3.76 (0.96)*
Please rate the extent to which you feel you have influence over work and work-related factors	4.00 (0.90)	4.27 (0.81)	+ 0.27*		4.42 (0.75)	3.45 (1.05)***

Item response scale: 1 = strongly disagree/very low, 2 = disagree/low, 3 = neither agree nor disagree/medium, 4 = agree/high, 5 = strongly agree/very high. Individual survey questions were not mandatory, so the reported *n* varies by item response

*SD *standard deviation

*p-value < 0.05, **p-value < 0.001, ***p-value < 0.0001, ^b^only asked on follow-up (post) survey, ^a^reverse coded

Additional survey items in the post-implementation survey assessed opinions regarding the effectiveness of training and communication surrounding the role as well as an early indication of how well the role has been working. These items scored on average between 3.73 (SD = 1.22) for the level of feedback staff were able to provide and 3.93 (SD = 1.04) for how well the goals of the new role were clearly communicated (see Additional file 1). The mean scores of the full sample of post-survey respondents was lower than the mean scores of matched sample respondents, indicating that the matched sample may have been more engaged or satisfied in the implementation efforts.

DCAs were surveyed to gauge their confidence in skills that are currently or would be required of them (Table 4). They reported significant improvements in their confidence in motivational interviewing (mean difference = + 0.85, *p* = 0.0017) and the ability to answer patients’ questions about their oral health (mean difference = + 0.55, *p* = 0.0007), whereas skills they were already proficient in (e.g., customer service, navigating referrals) remained highly rated. The skills DCAs reported feeling the least confident in post-implementation were explaining CAMBRA (mean = 2.63, SD = 0.90) and PEMBRA (mean = 2.59, SD = 0.89), the core risk-based caries and periodontal disease management protocols.

**Table 4:** Dental care advocates’ self-reported changes in skill confidence before and after training and implementation (*N* = 22)

Skills	Baseline (pre) mean (SD)	Follow-up (post) mean (SD)	Mean difference
Motivational interviewing	2.20 (1.15)	3.05 (0.94)	+ 0.85*
Care advocacy	2.65 (1.14)	3.30 (0.66)	+ 0.65*
Ability to answer patients' questions about their oral health care	2.80 (0.62)	3.35 (0.59)	+ 0.55**
Knowledge of oral health prescriptions and products	3.16 (0.60)	3.58 (0.51)	+ 0.42*
Knowledge of barriers patients face in improving their oral health	2.85 (0.59)	3.25 (0.72)	+ 0.40
Knowledge of resources, services, or tools to enhance patient care	3.10 (0.55)	3.45 (0.51)	+ 0.35*
Overall clinical dental knowledge	2.90 (0.72)	3.20 (0.70)	+ 0.30*
Triaging patients based on their oral health needs	3.15 (0.75)	3.40 (0.60)	+ 0.25
Navigating patients through referrals to specialty care	3.58 (0.51)	3.63 (0.50)	+ 0.05
Customer service	3.90 (0.31)	3.90 (0.31)	+ 0.00
Explaining the Proactive Dental Care Plan to patients^a^		3.07 (0.86)	
Explaining CAMBRA^a^		2.63 (0.90)	
Explaining PEMBRA^a^		2.59 (0.89)	
Explaining an effective therapeutic alliance^a^		2.68 (0.93)	

Item response scale: 4 = very confident, 3 = confident, 2 = somewhat confident, 1 = not at all confident. Individual survey questions were not mandatory, so the reported *n* varies by item response

*SD* standard deviation, *CAMBRA* Caries Management by Risk Assessment, *PEMBRA *Periodontal Management by Risk Assessment

*p-value < 0.05, **p-value < 0.001, ^a^question only asked on follow-up (post) survey

### Differences in opinions across demographics

**Fig. 2:**
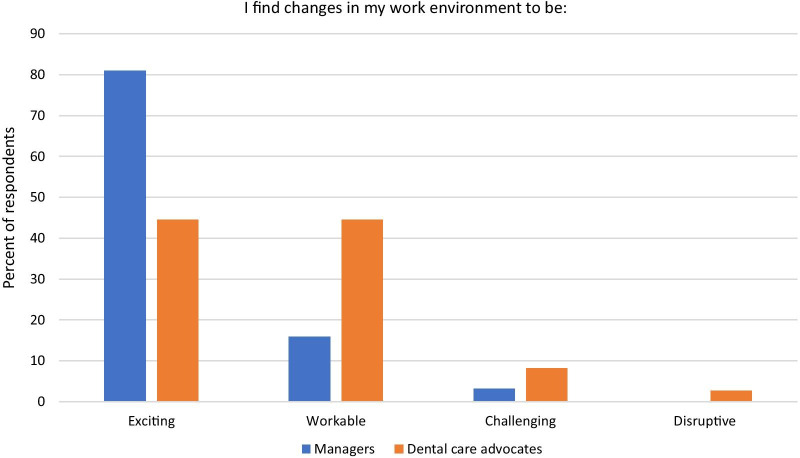
Follow-up beliefs in adaptive ability, by dental care advocates and managers

We stratified survey results by age, race/ethnicity, sex, or job type. Personal characteristics were not highly correlated with reported attitudes, except for consistent and statistically significant differences in attitudes and beliefs about the role post-implementation between DCAs and the management team (Table 3). These differences were significant and pronounced across all items except the ease of implementation (both groups found it moderately difficult). Like baseline survey results, all scores were above neutral, but DCAs themselves were less optimistic about the rollout compared to the practice managers and managing dentists. DCAs had more reserved opinions about the potential impact of the DCA role compared to managers, with the lowest reported mean being their belief in the role’s ability to improve workflow (3.41 vs. 4.37, *p* < 0.0001). The most statistically significant differences include the following: managers felt the DCA role was more important (4.86 vs. 3.96, *p* < 0.0001), understood the role better (4.85 vs. 4.21, *p* < 0.0001), and were more excited about implementing the role (4.75 vs. 3.76, *p* < 0.0001) compared to DCAs. Survey comments suggest that some DCAs thought the rollout was too quick, they did not feel sufficiently trained, and they were not given enough guidance on integrating the new roles and responsibilities into their existing duties. Figure 2 illustrates responses to one survey item about how adaptive they are to changes in their work environment: managers overwhelmingly report work changes to be exciting (81.0%) whereas less than half of DCAs similarly find changes to be exciting (44.5%). Instead, most DCAs find changes in their work environment to be workable (44.5%), challenging (8.2%), or even disruptive (2.7%).

Looking at other demographic differences in post-survey results (see Additional file 2), younger DCAs typically held less favorable attitudes and beliefs than older DCAs. There was no clear influence of age for managers, but one noticeable difference in complexity—managers over age 65 thought it would be more challenging to implement the change in roles compared to all other age brackets (65 + mean 2.33 vs. average mean 3.61). Hispanic/Latino/a/x DCAs universally reported more favorable attitudes and beliefs than did DCAs of all other racial/ethnic groups; however, non-Hispanic White managers reported the most positive attitudes and beliefs compared to managers of all other racial/ethnic groups. Male DCAs, though the sample of respondents was small, and managers nearly universally held lower opinions compared to female DCAs and managers.

## Discussion

This study describes the steps a large dental accountable care organization took to retool their existing front office staff to take on care coordination responsibilities and evaluates staff attitudes and the perceived benefits of the new DCA role within an implementation framework. Front desk staff are often overlooked health care personnel, but their core skill set in customer service lends itself well to an enhanced effort of patient engagement and care coordination. In the medical literature, care coordinators have been found to be key in defining quality in culturally responsive, patient-centered care [37], and their behaviors and attitudes are important factors in patient satisfaction and even treatment adherence [39, 40]. This study is one of the first to acknowledge and describe their strengths within the overall dental care team.

### Inner and outer settings for implementation

WDG is headquartered in Oregon, a state that has piloted and initiated progressive fiscally and socially responsible reforms of health care delivery and finance since the 1980s, a stark comparison to many other states that often turn to regressive, cost-cutting measures [27]. In 2012, Oregon transitioned nearly all of its Medicaid-insured population to Coordinated Care Organizations (CCOs). CCOs are regional managed care organizations with community oversight that are financially responsible for their members’ physical, mental, and dental health. Care coordination across these health domains is a key component of the CCO design, with metric and incentive structures in place to encourage more integrated coordination. WDG is highly engaged in statewide policy issues and is one of 9 original dental care organizations that contracted with CCOs to provide dental care to their members. The pilot project that stimulated the DCA role at WDG was one effort to integrate and coordinate care across one CCO’s medical and dental sectors.

As a dental accountable care organization, WDG is responsible for quality and total per capita costs across the full continuum of patient care, one of few examples of dental groups that practice under this model. In 2010, WDG re-trained their workforce to practice team-based dentistry: teams of clinicians (dentist, hygienists, and assistants) work synergistically in pods and are compensated by meeting quality metrics, not the volume of services provided [30]. Change occurs rapidly and frequently at WDG, and staff are generally supportive of the organization’s progressive nature [29, 35]. The implementation culture at WDG is top-down, with the executive team’s visions disseminated across the company by a core group of directors and managers who create guidelines, training programs, and implementation strategies. Bringing the front office staff on as a team member, not just figuratively but with critical responsibilities to support the patient’s clinical care, aligns well with WDG’s strong commitment to practicing team-based dentistry. The new role also augments WDG’s mission to practice patient-centered care by providing high-risk patients a dedicated advocate on the clinical care team.

Policy drivers in the outer setting stimulated care coordination implementation, which was then greatly facilitated through capacity for change in WDG’s inner setting. The interface between these two settings is dynamic, and these settings richly described the impetus and context for change. The pilot project provided the evidence base and stimulated implementation company-wide, effectively facilitated through the progressive, mission-driven company culture and eager executive buy-in. Apparent and strong drivers for implementation paired with a history of similar, successful interventions set the positive implementation environment for the DCA role.

### Implementation process

A dedicated leadership team and centralized system for executing the process in a stepwise and organized fashion facilitated the quick implementation process. This new role’s fast-paced implementation was well received as evidenced by positive staff attitudes and beliefs. Similarly, appointing implementation leaders and champions within each practice facilitated the spread of the general enthusiasm for the new role; however, the initial plan to allow clinics to determine best practices in role and workflow modifications was not as readily accepted as the more structured description unveiled at the company-wide training, requiring a revised implementation plan. Sticking to the organization’s strengths in executing top-down interventions with robust centralized planning and oversight seemed to be the better approach for the role’s implementation in this setting.

### Characteristics of individuals

There was strong buy-in from executive and managerial teams to support implementation and high staff acceptance and enthusiasm for the DCA role before adoption. However, differences were seen in post-implementation opinions of the role, pointing to areas of further refinement. The mandatory transition from front office personnel to DCAs understandably appeared to be more challenging for the DCAs themselves, the staff tasked with taking on new responsibilities, and they may need extra training or support to successfully transition from front office personnel to certified DCAs. Despite these differences of opinions, the staff was overwhelmingly excited about being on the forefront of bringing care coordination to dentistry.

### Study limitations

Limitations to this study include relatively small sample sizes for the surveys, although we were able to observe statistically significant differences on several items, suggesting our power was sufficient. It is possible that selection bias was created by the 61.3–69.0% response rates, with only people with a positive view participating in the surveys; however, there was variation in the ratings, suggesting that participants were willing to give comparatively higher or lower ratings for different items. There could be hesitance from participants to share negative views of the program, fearing it may affect their professional standing within the organization, but we tried to minimize this by having outside researchers (not WDG) inviting staff to participate and having anonymous surveys. Finally, findings are representative of just one large dental organization in the US, and implementation of similar efforts in other settings may face different challenges. Care should be taken when generalizing these results outside of this kind of practice model, and further implementation studies with other practice models are needed.

### Future research

The initial transformation of front office personnel to DCAs was complete and successful in training and deployment, but the long-term outcomes are not yet known. Future work building from this implementation study will evaluate long-term adoption of care coordination, additional work around enhancing team-based dentistry, and how this adoption has influenced patient care and health behaviors and outcomes from the patient perspective.

## Conclusions

This study examined the implementation of care coordination in a specific dental organization in the US utilizing their existing front office workforce. In addition to their administrative roles, office staff are now upskilled to be the new DCA workforce. Dental managers were extremely positive about the new role, while DCAs had positive impressions but were less enthusiastic. With the strong innovative culture and centralized administrative structure at WDG, paired with high staff excitement and satisfaction with the new role, initial implementation was successful.

Care coordination serves as a critical mechanism for the management of populations, greatly facilitating advancements in care delivery, including value-based payment and the move to integrated care delivery systems. As systems move toward integrated care across medicine, behavioral health, and dentistry, these roles will be even more important to truly coordinate care across these sectors. The globalization of risk-based care management requires significant retooling of the workforce to enable success. This is one example of how an already progressive and team-based model of dentistry has effectively adapted existing personnel to provide coordinated care to manage risk-based patient populations. The knowledge gained from studying care coordination implementation in this example has the potential to inform and support additional efforts to utilize care coordination in dentistry.

## Supplementary Information

**Additional file 1.** Full sample pre- and post-survey results. Contains a table of each survey item, respondents for each item, and item means and standard deviations for both the baseline and follow-up surveys. Table legend: SD = standard deviation; ^†^ reverse coded; ^‡^ only asked on follow-up (post) survey; Individual survey questions were not mandatory, so the reported *n* varies by item response.

**Additional file 2.** Post-survey results (means) by demographics and role. Contains a table of each post-survey item with mean responses stratified by job type (dental care advocates, managers), age group, race/ethnicity, and gender. Table legend: ^†^ only asked on follow-up (post) survey; ^‡^ reverse coded; DCA = dental care advocate; AI/AN = American Indian/Alaska Native; ND = insufficient data to report; Individual survey questions were not mandatory, so the reported *n* varies by item response.

## Data Availability

The datasets used and/or analyzed during the current study are available from the corresponding author on reasonable request.

## Abbreviations

CAMBRA: Caries Management by Risk Assessment
CCO: Coordinated Care Organization
CFIR: Consolidated Framework for Implementation Research
DCA: Dental care advocate
EHR: Electronic health record
PEMBRA: Periodontal Management by Risk Assessment
SD: Standard deviation
WDG: Willamette Dental Group
USD: United States dollars
